# Human development index in a context of human development: Review on the western Balkans countries

**DOI:** 10.1002/brb3.1755

**Published:** 2020-08-09

**Authors:** Boban Dasic, Zeljko Devic, Nebojsa Denic, Dragan Zlatkovic, Ivana D. Ilic, Yan Cao, Kittisak Jermsittiparsert, Hiep Van Le

**Affiliations:** ^1^ Faculty of Trade and Banking Alfa BK University Belgrade Serbia; ^2^ College of Economics Pec‐Leposavic Leposavic Serbia; ^3^ Faculty of Sciences University of Pristina Kosovska Mitrovica Serbia; ^4^ Faculty of Mathematics and Computer Sciences Alfa BK University Belgrade Serbia; ^5^ Department for Mathematics and Informatics Medical Faculty University of Nis Nis Serbia; ^6^ School of Mechatronic Engineering Xi’an Technological University Xi’an China; ^7^ Department for Management of Science and Technology Development Ton Duc Thang University Ho Chi Minh City Vietnam; ^8^ Faculty of Social Sciences and Humanities Ton Duc Thang University Ho Chi Minh City Vietnam; ^9^ Institute of Research and Development Duy Tan University Da Nang Vietnam

**Keywords:** developmental level, education, human development index, international comparisons, living standards

## Abstract

**Introduction:**

The Human Development Index (HDI), as one of the more complex composite indicators of the level of human potential and quality of life, is a combination of three dimensions (indicators, factors): life expectancy at birth, the middle number of years of education and the expected number of years of schooling combined into a single education index and economic benefits expressed by production, or GDP (gross domestic product) according to purchasing power (PPP US $).

**Methods:**

The same measures and average achievements in the field of health, education, and living standards are presented. The HDI was first developed in 1990 under the United Nations Development Program (UNDP) and is published as Human Development Reports (HDR). At present, it has become the most widely used complex indicator suitable for international comparisons and assessments of the achieved development level of a particular country or region.

**Results:**

The paper deals specifically with the more perspective view of human development in the Western Balkans, with a series of socio‐economic implications for the development policy of the countries under observation.

**Conclusion:**

The particular significance of the conducted research stems from the fact that in the countries of the Western Balkans are identified factors at the beginning of the transition period were often marginalized in the creation of macroeconomic policies, but in recent years there have been more positive developments in that regard.

## Graphical abstract

The Human Development Index (HDI)—the level of human potential and quality of life. Measures in the field of health, education, and living standards are presented. Factors at the beginning of the transition period were often marginalized

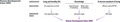

## 1 INTRODUCTION

The wealth of a state is made up of people. The primary goal of development is the creation of such an environment that will enable people for a long, healthy, and creative life (UNDP, 1990). Human development is the extending process of people's choices. One can say this is a process of increasing the significance of human values. Naturally, it is a complex phenomenon that has been seen in different aspects—demographic, cultural, political–legal, and socio‐economic. Based on this phenomenon, some estimates are made of its impact on the economic and national development of the country. The national event is in correlation with human potential. The complexity of this relationship best illustrates the view that there is no simple answer to the simple question of whether nations are rich because they are better educated or better educated because they are rich (Blaug, 1976).

The human development paradigm emphasizes two simultaneous processes: The building of human abilities and how people use them to function in society and make choices between options that they have in all aspects of their lives (UNDP, 2018a). The phenomenon of human development, which takes into account the close links between economic, social, cultural, spatial, educational, and healthcare, encompasses a safe economy, adequate nutrition, environmental protection, personal safety, community security, and broader political security. Current and future generations must be aware of their responsibilities when it comes to development. Personal and social security should be sought if it enables a decent life, in an economy where profit is distributed equally to all, and not only to a few and the environment whose fruits and pleasure can be used without fear. This concept provides a long and healthy life people.

The world is characterized by dynamic processes and significant changes in the overall social, political, economic and social environment, determining, and multiplying developmental specifics. Positive changes result in a better opportunity for people's lives, longer life expectancy, and better education, while adverse changes create developmental problems. It is important to emphasize that development problems cannot be explained exclusively by economic indicators. The process of measuring and interpreting differences in development is a much more complex problem. Measuring growth in a new globalized world requires a shift from the economic and to the noneconomic sphere (social and society). Development indicators should give a more realistic picture of the economic progress of a particular country. Only in this way, economists will identify the underlying development problems, offering suggestions to macroeconomic policymakers how to act in certain situations.

Development is in most of its conceptual history, portrayed as the normal process of change, or as a quest for economic growth. By the beginning of the nineties, the GDP was routinely used as the only indicator of the achieved level of development. After that, a series of new signs are emerging that are more comprehensive, multi‐dimensional, and from some different aspects, looking at events in the growth and development of an economy (Potter, Binns, Elliott, Nel, & Smith, 2018).

Since 1990, the United Nations Development Program (UNDP) has been implementing a human development program by applying an approach that is not confined to national income alone but is focused on people and their ability to achieve the full potential to lead a healthy, productive and creative life. The first human development report published in 1990, “People are the real wealth of nations,” began a new approach to thinking about development (Ferjan, 2014). To date, 26 Human Development Reports (HDRs) have been published, which are the result of the calculation of the Human Development Index (HDI) for each country, based on which the ranking of countries in the world is carried out. The HDI is a widely cited statistic that is commonly used as a measure of well‐being in different countries (Engineer, King, & Roy, 2008).

In this paper is presented perspective view of human development in the Western Balkans, with a series of socio‐economic implications for the development policy of the countries under observation. The main significance of the research stems from the fact that in the countries of the Western Balkans are identified factors at the beginning of the transition period were often marginalized in the creation of macroeconomic policies, but in recent years there have been more positive developments in that regard.

## 2 METHODOLOGY

Analyzing the entire spectrum of indicators in HDI assesses progress in achieving many aspects of human development (Republicki zavod za razvoj, 2007). According to the UNDP methodology, in the period from 1990, when it officially began to apply, and until 2010, the HDI contained a combination of three different indicators:

1. General quality of life, expressed by the expected duration of life;
2. Literacy, measured by a combination of two indicators: the literacy rate of the adult population (weighted by 2/3 significance) and the total enrollment rate in primary, secondary, and higher education (weighted by 1/3 of the character);
3. The standard of living, that is, economic benefits expressed by production, that is, GDP (gross domestic product) in terms of purchasing power (PPP US $). The analysis of purchasing power parity allows seeing the differentiation in purchasing power between countries by eliminating differences in the price level. It is most commonly used in international comparisons of GDP and its components. The program of monitoring and comparison of purchasing power parity purchasing power at the international level is under the responsibility of EUROSTAT's statistics, which publish annually the Purchasing Power Parities Report (https://ec.europa.eu/eurostat/web/purchasing‐power‐parities) for a period of three years, including by comparing and comparing the prices for about 3,000 comparative and representative products that enter the composition of GDP of the OECD countries, based on which the relative price level of each state is determined in relation to the OECD average.

The above three indicators, used to calculate HDI, are available in almost all international statistical anniversaries and relate to the quality of life achieved in terms of life expectancy, literacy and accessibility of the school system to the individual. Conducting these three indicators to one common measure is done by setting a minimum, equal to "0" and a maximum, equal to 1 for each dimension. Each of these indicators is weighted with the relative share in the total number of signs. A set of weighted indicators creates a complex HDI and determines the position for each country on a scale of 0–1 (0 < HDI > 1).

Hence, HDI is a simple arithmetic mean of all three primary indices:

(1) $$\text{HDI}=\frac{I_{1}+I_{2}+I_{3}}{3}$$

where *I*_1_ represents the life expectancy index, *I*_2_ education index, and *I*_3_ GDP index.

All three primary indices are standardized according to the principle.

(2) $$\frac{\left(I‐I_{\min}\right)}{\left(I_{\max}‐I_{\min}\right)}$$

where “*I*” represents the actual value in the country.

The minimum and maximum values of individual indices are listed in Table 1.

**Table 1:** Summary of HDI reform (Jakopin, 2010)

Dimensions	Previous (1990–2010)			From 2010		
	Indicators	Transformation		Indicators	Transformation	
		Min.	Max.		Min.	Max. (detected values)
Health	Life expectancy at birth (year)	25	85	Life expectancy at birth (year)	20	83.2
Knowledge (education)	Adult literacy rate (%)	0	100	Expected number of years of schooling	0	20.6
	Combined gross registration rate (%)	0	100	Average number of years	0	13.2
Living standards	GDP per capita (PPP US$)	100	40,000 (limited)	GDP per capita (PPP US$)	163	108.211
Aggregation	Arithmetic mean			Geometric mean		

By 2010, all the countries of the world were classified into one of three groups, which indicate the level of human development achieved:

- 0.00 < HDI < 0.50 – low level of human development;
- 0.50 < HDI < 0.80 – medium level of human development;
- 0.80 < HDI < 1.00 – high level of human development,

and now they are classified into the following groups:

- 0.00 < HDI < 0.55 – low level of human development;
- 0.55 < HDI < 0.70 – medium level of human development;
- 0.70 < HDI < 0.80 – high level of human development and
- 0.80 < HDI < 1.00 – very high level of human development (http://www.hdr.undp.org/sites/default/files/2018_human_development_statistical_update.pdf).

The purpose of calculating the HDI is to rank global economies by the level of HDI and to compare such a ranking with those that are exclusively based on the GDP per capita (PPP US $). Three cases are possible:

1. If the HDI rank is close to GDP per capita (PPP US $) ranking, it means there is a harmony between existing resources and development results.
2. If the HDI rank is higher than the GDP per capita (PPP US $) rank, it means that these areas have used their potentials in the best possible way, that is, development policy is in the function of the entire population.
3. If the HDI rank is lower than the GDP per capita (PPP US $) rank, it means that the allocation of resources in the best possible way; that is, their policy of development is not in the function of the entire population, but favors the ruling classes (oil‐exporting countries and similar economies based on the exploitation of natural resources and the mono‐cultural economy based on them).

**Figure 1:**
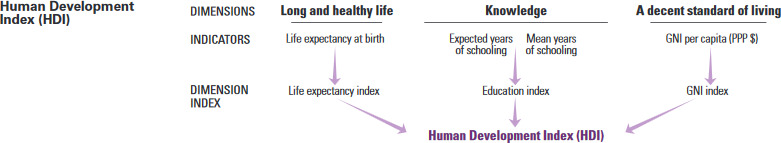
Calculating the human development indices—graphical presentation (http://hdr.undp.org/sites/default/files/hdr2018_technical_notes.pdf)

At 2010, the HDI experienced some changes in the calculation of individual idioms (Figure 1).

The access to knowledge has undergone some changes, and it is measured through:

- the average number of years of education among the adult population, which represents the average number of years of schooling that the community of 25 or more years of age has acquired during life, and
- The expected number of years of education for children at the time of enrollment in school, which is the total number of years of schooling scheduled for children at the time of admission in school, provided that existing forms of enrollment rates for specific ages remain the same throughout the life of the child.

Changing the calculating methodology of the "access to knowledge" indicator was necessary because a number of countries, especially those at the top of the HDI scale, reached high levels of combined gross enrollment and adult literacy rates, which made the relevance of these indicators weaker, and new indicators better reflected the concept of education rather than the previous and more precisely indicate the differences between the countries. The lack of past and present indicators of “access to knowledge” is that neither one nor the others assess the quality of education.

The living standard as the third indicator of HDI has changed and is now measured by the gross national income per capita expressed in constant international dollars for 2011 converted using the purchasing power parity rate—GDP per capita (PPP US $). GDP does not reflect the available national income (some profits may be repatriated abroad, some residents of the country receive remittances from abroad, and in some cases, financial assistance entering the country can be significant), and GDP adjusts GDP for these factors and, therefore, is better a measure of the country's income level.

According to the previous method, the HDI was calculated as the mean arithmetic value of the dimension indicator, allowing the substitution among the dimensions, so that high achievements in other sizes could offset the more unfortunate results in one aspect. A multiplicative aggregation method is now in use, where aggregations are made using the geometric mean value of dimension indicators, which reduces the level of interchangeability between dimensions and ensures that a reduction of 1% in, for example, the GDP per capita (PPP US $) has the same impact on the HDI as a 1% drop in education or life expectancy.

HDI calculation is done in two steps. The first step is to create a dimension indicator. The dimension indicators that are measured in different units are transformed into a scale with no groups ranging from 0 to 1, by setting the minimum and maximum values for each indicator, which are also listed in Table 1.

The UNDP experts’ team in selecting the lowest value indicators has been guided by the principle of survival or "natural zero" below which there is no chance for human development. Maximum values are the highest values in the observed time series (1980–2013).

The dimension indicators are calculated as follows (Klugman, Rodríguez, & Choi, 2011):

(3) $$H_{h}=\frac{\left(l_{e}‐l_{e_{\min}}\right)}{\left(l_{e_{\max}}‐l_{e_{\min}}\right)}$$

(4) $$H_{e}={\left[\left(\frac{\left(\text{mys}‐{\text{mys}}_{\min}\right)}{\left({\text{mys}}_{\max}‐{\text{mys}}_{\min}\right)}\right)*\left(\frac{\left(\text{eys}‐{\text{eys}}_{\min}\right)}{\left({\text{eys}}_{\max}‐{\text{eys}}_{\min}\right)}\right)\right]}^{\frac{1}{2}}$$

(5) $$H_{1s}=\frac{\left(\ln\left(\text{gni}\right)‐\ln\left({\text{gni}}_{\min}\right)\right)}{\left(\ln\left({\text{gni}}_{\max}\right)‐\ln\left({\text{gni}}_{\min}\right)\right)}$$

*H*_h_ represents the life expectancy index, *H*_e_ index of education, *H*_ls_ index of living standard, *l*_e_ real‐life expectancy, mys real value of the middle number of years of schooling, eys expected some years of education; *l*_n_ is the natural logarithm, and gni the value of GNI(PPP US$).

The second step is to collect the dimension indicators for the HDI calculation.

(6) $$\text{HDI}={\left(H_{\text{Health}}*H_{\text{Education}}*H_{\text{Living}\;\text{standard}}\right)}^{\frac{1}{3}}$$

Transformed by the use of these minimums and maxims, the HDI provides an aggregate measure of the achievement of the human development of a country concerning what it is at the moment sustainable.

To ensure the highest level of comparability among countries, the HDI is based on international data which for the following types of indicators:

- Life expectancy at birth: United Nations Department of Economic and Social Affairs – UNDESA (https://www.un.org/development/desa/en/).
- Expected years of schooling: UNESCO Institute for Statistics (http://www.unesco.org/new/en/natural‐sciences/science‐technology/overview‐of‐unescos‐work/unesco‐institute‐for‐statistics/). ICF Macro Demographic and Health Surveys (https://www.icf.com/resources/projects/research‐and‐evaluation/demographic‐and‐health‐surveys), United Nations Children's Fund (UNICEF) Multiple Indicator Cluster Surveys (http://mics.unicef.org/) and OECD (http://www.oecd.org/).
- Mean years of schooling: UNESCO Institute for Statistics (Barro & Lee, 2016), ICF Macro Demographic and Health Surveys, UNICEF Multiple Indicator Cluster Surveysand OECD.
- GNI per capita: World Bank (https://www.worldbank.org/), IMF (https://www.imf.org/external/index.htm) and United Nations Statistics Division (https://unstats.un.org/home/).

Comparing values and ranking in the latest Human Development Report 2018 with values and ranks from previously published reports by 2010 is not recommended, due to changed methodology, revision, and updating of primary data and adjustment of limit values.

In the latest Human Development Report 2018, the HDI indices for the period 1990–2017 comprise the compilation of data between countries, as well as tracking trends from the previous period. The latest HDI and ranking data are based on consistent indicators, methodology and time series data, which provide an overview of real changes in values and ranking over time, reflecting the real shift that the countries have made. The HDI's trends present essential facts at the national, regional, and global levels, highlighting substantial differences both in welfare and in life opportunities among countries over the years.

The HDI value, ranging from 0 to 1, shows the country that has reached that country's reach to its maximum value, which allows comparisons with other countries. The difference between the achieved and the maximum possible HDI value is aimed at showing the shortcomings of that country, with the challenge for each state to find ways to reduce these deficiencies, that is, to bring them as close as possible to the maximum value.

Although HDI is an indicator that ranks countries toward the level of human development, it will correctly never include social development in its full sense (Kovacevic, 2011).

There is no need for an Ethical approval in this study since there are no human participants.

### 2.1 HDI value in the world and by groups of countries

In the latest human development report, Human Development Indices and Indicators 2018 Statistical Update, the world's highest HDI value is 0.728, and is classified in countries of human development for countries of very high human development HDI 0.894 for countries of high human development 0, 757 for states of middle human development 0.645 and for low human development countries 0.504. Table 2 shows the HDI values for the world and human development groups, as well as information regarding HDI elements.

**Table 2:** Values of HDI and its indicators by groups of countries and in the world (UNDP, 2018b)

Human development groups	Human Development Index (HDI) Value	Life expectancy at birth (years)	Expected years of schooling (years)	Mean years of education (years)	Gross national income (GNI) per capita (2011 PPP $)
World	0.728	72.2	12.7	8.4	15,295
Very high human development	0.894	79.5	16.4	12.2	40,041
High human development	0.757	76.0	14.1	8.2	14,999
Medium human development	0.645	69.1	12.0	6.7	6,849
Low human development	0.504	60.8	9.4	4.7	2.521

The latest HDR shows HDI values for 189 countries. In the group of countries with a very high HDI value, there are 59 countries (Table 3).

**Table 3:** Human Development Index and its components – the first ten countries belonging to the group Very High Human Development (UNDP, 2018c)

HDI rank		Human Development Index (HDI) Value	Life expectancy at birth (years)	Expected years of schooling (years)	Mean years of education (years)	Gross national income (GNI) per capita (2011 PPP $)	GNI per capita rank minus HDI rank	HDI rank
		2017	2017	2017	2017	2017	2017	2016
1.	Norway	0.953	82.3	17.9	12.6	68.012	5	1
2.	Switzerland	0.944	83.5	16.2	13.4	57.625	8	2
3.	Australia	0.939	83.1	22.9	12.9	43.560	18	3
4.	Ireland	0.938	81.6	19.6	12.5	53.754	8	4
5.	Germany	0.936	81.2	17.0	14.1	46.136	13	4
6.	Iceland	0.935	82.9	19.3	12.4	45.810	13	6
7.	Hong Kong, China	0.933	84.1	16.3	12.0	58.420	2	8
8.	Sweden	0.933	82.6	17.6	12.4	47.766	9	7
9.	Singapore	0.932	83.2	16.2	11.5	82.503	−6	8
10.	Netherlands	0.931	82.0	18.0	12.2	47.900	5	10

The country with the highest HDI is Norway (0.953), followed by other countries with lower HDI values. It does not necessarily mean that while Norway is at the top of the HDI value, it also has the best benefits of indicators that make up HDI. Thus, for example, can see that the first 10 countries observed for Life expectancy at birth Hong Kong have the highest value (84.1), for Expected years of schooling Australia (22.9), for Mean years of schooling Germany (14.1), while for Gross national income (GNI) per capita is Singapore's leading (82,530) (Table 4).

**Table 4:** Human Development Index and its components – Low Human Development (Grimm et al., 2008)

HDI rank		Human Development Index (HDI) Value	Life expectancy at birth (years)	Expected years of schooling (years)	Mean years of education (years)	Gross national income (GNI) per capita (2011 PPP $)	GNI per capita rank minus HDI rank	HDI rank
		2017	2017	2017	2017	2017	2017	2016
187.	South Sudan	0.388	57.3	4.9	4.8	963	−1	186
188.	Central African Republic	0.367	52.9	7.2	4.3	663	3	187
189.	Niger	0.354	60.4	5.4	2.0	906	−2	188

The group of countries with high‐value HDI includes 52 countries, including Croatia (0.838) and 50th place (0.814) in the Western Balkans.

The Mid‐HDI countries group includes 38 countries, and it contains all the remaining countries of the Western Balkans, Serbia (0.787) in 67th place, Albania (0.785) in 68th place, Bosnia and Herzegovina (0.768) in 77th place and FYR Macedonia (0.757) in 80th place.

The low HDI group consists of the remaining 37 countries out of a total of 189 countries. The worst‐ranked countries are South Sudan (0.388), Central African Republic (0.367), and finally Nigeria (0.354) in the last 189th place.

Of the total number of countries surveyed (189), the highest life expectancy at birth as a component of HDI is Hong Kong (84.1), which is HDI at 7th place; Japan (83.9) in 19th place and Switzerland (83.5) at place number 2. For Expected years of schooling, the highest values are Australia (22.9), which is HDI in 3rd place; Belgium (19.8) in 17th place and Ireland (19.6) in 4th place. For the Mean years of schooling, the highest values are Germany (14.1), which is HDI in the 5th place; Switzerland (13.4) in 2nd place and USA (13.4) in 13th place. For Gross national income (GNI) per capita, the highest values are Qatar (116,818), which is HDI at 37th place; Liechtenstein (97,336) in 17th place and Singapore (82,503) in 9th place. From the above, it can be seen that countries that have the highest HDI values do not have the highest amounts of individual indicators that are integral to the HDI. Their leading HDI values are precisely the composite measure of the achievements of these indicators that are an essential part of the HDI.

## 3 ANALYSIS OF HDI TRENDS IN THE PERIOD 1990–2017

**Figure 2:**
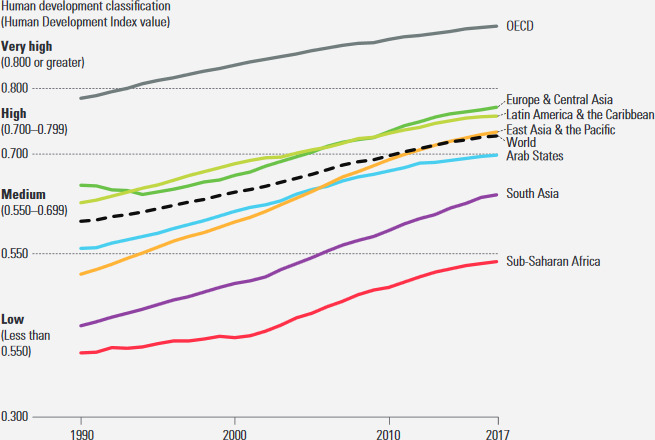
Human Development Index values, by country grouping, 1990–2017 (UNDP, 2018e)

Comparing the HDI values by years (1990, 2000, 2010, 2012, 2014, 2015, 2016 and 2017), a linear increase in the HDI value can be observed. At the global level, from the beginning of the introduction of the HDI to the end of 2017 (HDI 0.728), we have an increase of 21.7% compared to 1990 (0.598) (Table 5). This growth for countries belonging to the Very High Human Development is 12.5%, for the High Human Development countries 32.6%, for Medium Human Development countries 39.6% and Low Human Development countries 10.2%. HDI growth rates vary by state. It can be concluded that the countries that belong to the Medium Human Development group have achieved the highest growth, but this growth is insufficient to transform them into more development HDI levels (Figure 2).

**Table 5:** Human Development Index Trends, 1990–2017 (UNDP, 2018d)

World/Human development groups	Human Development Index (HDI) – Value							
	1990	2000	2010	2012	2014	2015	2016	2017
World	0.598	0.642	0.698	0.709	0.718	0.722	0.726	0.728
Very high human development	0.787	0.831	0.873	0.880	0.887	0.890	0.892	0.894
High human development	0.571	0.635	0.718	0.732	0.745	0.750	0.754	0.757
Medium human development	0.462	0.523	0.596	0.613	0.627	0.634	0.641	0.645
Low human development	0.351	0.387	0.472	0.468	0.495	0.498	0.501	0.504

From the above chart, it can be noted that in the period 1990–2017. The growth of the world HDI was 21.7%. South Asia was the fastest growing region with 45.3%. East Asia and the Pacific follow it by 41.8% and sub‐Saharan Africa with 34.9%. The countries of Sub‐Saharan Africa are still in the low human development group, although they have approached the Medium Human Development group. South Asia is a member of the Medium Human Development Group, while East Asia and the Pacific are in the period 1990–2017 moved from Low Human Development to a group of countries with High Human Development. States of the Organization for Economic Co‐operation and Development (OECD) recorded an increase of HDI in the mentioned period by 14.0%. This growth rate is lower than the growth rate of the countries listed, but it should be noted that the OECD countries are in the very high human development group and are approaching the maximum value of HDI. In particular, it should be kept in mind that different HDI components have their limits. There is a biological limit of life expectancy, and years of schooling and enrollment rates cannot grow unlimited, while income is the only integral part of the HDI that could continue to grow, but revenue growth slows down as the economy mature. It is important to note that the amount of 75,000 dollars per capita has been designated as an upper limit because it has been demonstrated that it practically does not benefit from human development and well‐being from annual income per capita above $ 75,000 (Kahneman & Deaton, 2014).

Factors that caused lower HDI growth rates in the period 1990–2017 are various armed conflicts in some countries and regions (for example, Libya, which ranks 82nd in HDI in 2012 to 108th in 2017, the Syrian Arab Republic from 128th place in 2012) to 155th in 2017, Yemen from 158th place in 2012 to 178th place in 2017), various epidemics (HIV/ AIDS in Sub‐Saharan Africa caused a dramatic decline in life expectancy), natural catastrophes, climate change, or economic crisis (the 2008 World Economic Crisis, hyperinflation, the introduction of market mechanisms in postsocialist countries a, oscillations in food prices, etc.). Due to the fact of the group as mentioned above of factors, some countries suffered severe losses, losing in the years that everything has been done for decades. There are 1,650 million poor in the world living in poor living conditions (short life expectancy), without access to education and health care systems (Alkire & Santos, 2010).

One of the major threats to social development is a long‐term vulnerability. If we remove the causes of weakness, then everyone will be able to participate in advancement, which will make social development more just and sustainable (UNDP, 2014).

Despite these challenges, countries in these regions have recovered from the losses caused by these factors.

Table 6 shows the annual HDI growth in the world and by the groups of countries. It is noted that the countries that belong to the Low Human Development group had the highest increase.

**Table 6:** Average annual HDI growth – % (UN, 2017)

World/Human development groups	Average annual HDI growth – %			
	1990–2000	2000–2010	2010–2017	1990–2017
World	0.72	0.84	0.60	0.73
Very high human development	0.55	0.50	0.34	0.48
High human development	1.06	1.24	0.76	1.05
Medium human development	1.25	1.32	1.13	1.24
Low human development	1.00	1.99	0.93	1.35

Observing the increase in HDI ranking by countries in the period 2012–2017 the highest increase was recorded in Ireland (progress for 13 places), and Botswana, the Dominican Republic and Turkey (progress for eight positions). The most significant drop was recorded by the Syrian Arab Republic (fall by 27 places), Libya (fall by 26 places) and Yemen (fall by 20 places) (UNDP, 2018f).

### 3.1 Trends of HDI country index in the Western Balkans

In this part of the paper, a comparative analysis of the HDI of the Western Balkan countries was made, namely Croatia, which is a member state of the European Union and Montenegro, Serbia, Albania, Bosnia, and Herzegovina and FYR Macedonia that are not yet European Union member states.

From the countries of the Western Balkans, Croatia, and Montenegro fall under the category Very high human development. Their HDI is below the average for the specified group of countries to which they belong. Serbia, Albania, Bosnia, and Herzegovina have HDI above the average for that group of countries to which they belong, while FYR Macedonia has HDI which is the same as the average for this group of countries (Table 7).

**Table 7:** Human Development Index and its components – Western Balkans (UNDP, 2018g)

HDI rank		Human Development Index (HDI Value	Life expectancy at birth (years)	Expected years of schooling (years)	Mean years of education (years)	Gross national income (GNI) per capita (2011 PPP $)
46.	Croatia	0.838	77.8	15.0	11.3	22,162
50.	Montenegro	0.814	77.3	14.9	11.3	16,779
67.	Serbia	0.787	75.3	14.6	11.1	13,019
68.	Albania	0.785	78.5	14.8	10.0	11,886
77.	Bosnia and Herzegovina	0.768	77.1	14.2	9.7	11,716
80.	The former Yugoslav Republic of Macedonia	0.757	75.9	13.3	9.6	12,505

Croatia has the highest HDI value (0.838), which ranks 46th out of the total number of 189 countries for which the index is measured. Croatia is in the observed group of countries, in almost all indicators that measure HDI, at the very top in terms of their value. The outcome is an indicator Life expectancy at birth where Albania has a higher value than Croatia (77.1:77) and an indicator of the Mean years of schooling where Croatia and Montenegro have the same amount. The above data point to a better international position of Croatia when taking into account other dimensions that make the quality of life apart from purely material (Borozan, Drvenkar, & Savić, 2016). Although Croatia is a member of the European Union and in terms of GDP per capita (PPP US $) significantly ahead of other Western Balkan countries that are not yet members of the European Union, this does not mean that other countries of the Western Balkans cannot have a higher value and better rank HDI, that is to say, Croatia in the ranking list. In a study by Konya and Guisan, it has been confirmed that some underdeveloped countries have managed to increase the value and ranking of HDI concerning individual developed countries (Konya & Guisan, 2008).

Montenegro is in the 50th position according to HDI (0.814). From the observed group of countries, it is found in all indicators behind Croatia, except for the Mean years of schooling indicator where they are equal. According to the value of BND per capita (PPP US $), Montenegro is best positioned by the observed group of countries that are not members of the European Union (16,779).

Serbia has an HDI of 0.787, ranking 67th out of 189 countries. Of all the HDI indicators, Serbia has the lowest life expectancy at birth (75.3) of the observed group of countries. With the value of Gross national income (GNI) per capita (13,019), Serbia is among the middle‐income countries. Considering that growth in investment in education is projected, with the simultaneous growth of other factors that constitute HDI, it is also expected that Serbia's ranking on the HDI ranking will be expected.

Albania is at the heart of Serbia's HDI. Albania has the highest life expectancy at birth (78.5) of the observed group of countries.

Bosnia and Herzegovina are HDI (0.768), better positioned (77th) than FYR Macedonia (80th) whose HDI is the lowest of the observed group of countries (0.757). The value of Gross national income (GNI) per capita of Bosnia and Herzegovina (11,716) is the lowest of all countries of the Western Balkans.

FYR Macedonia is the worst‐ranked Western Balkan country (80th) in terms of HDI. The weakest values of the indicators that make up the HDI of FYR Macedonia concerning the other countries of the Western Balkans relate to the Expected Years of Schooling and Mean Years of Schooling (Table 8).

**Table 8:** Human Development Index trends, 1990–2017 (UNDP, 2018h)

	Croatia	Montenegro	Serbia	Albania	Bosnia and Herzegovina	The former Yugoslav Republic of Macedonia
HDI 1990	0.670	/	0.718	0.645	/	/
HDI 2000	0.750	/	0.711	0.669	0.672	0.669
HDI 2010	0.808	0.793	0.759	0.741	0.713	0.735
HDI 2012	0.816	0.800	0.768	0.767	0.739	0.740
HDI 2014	0.824	0.805	0.775	0.773	0.754	0.747
HDI 2015	0.827	0.809	0.780	0.776	0.755	0.754
HDI 2016	0.828	0.810	0.785	0.782	0.766	0.756
HDI 2017	0.831	0.814	0.787	0.785	0.768	0.757
Change in HDI rank 2012–2017	0	0	0	0	7	2
Average annual HDI growth % 1990–2000	1.14	/	−0.11	0.37	/	/
Average annual HDI growth % 2000–2010	0.75	/	0.66	1.02	0.60	0.94
Average annual HDI growth % 2010–2017	0.40	0.36	0.52	0.83	1.07	0.42
Average annual HDI growth % 1990–2017	0.80	/	0.34	0.73	/	/

Based on the above analysis of HDI countries in the Western Balkans, we can conclude that inequality in income is generally higher than inequality in education and life expectancy. Similar conclusions were reached by Grimm, Harttgen, Klassen, and Misselhorn in a 2008 survey (Grimm, Harttgen, Klasen, & Misselhorn, 2008).

## 4 DISCUSSION

Looking at the HDI for the countries of the Western Balkans by years, it can be seen as gradual growth. Highest growth of HDI rankings in the period from 2012 to 2017 Bosnia and Herzegovina (an increase of 7 seats), then FYROM (increase for two places), while other countries of the Western Balkans retained their positions. In the period 1990–2017, Croatia (0.80%), Albania (0.73%), and Serbia (0.34%) achieved the highest average HDI growth. If such a trend of growth continues, it can be expected that Serbia, Albania, Bosnia and Herzegovina and FYR Macedonia will move from the High Human Development group to the Very High Human Development for 10–15 years.

In the 1980s and 1990s, the Western Balkan countries, sometime later, started the process of transition. The collapse of the socialist system and the economic planning process left great consequences for the group of countries mentioned. The development implied the implementation of reforms that are, among other things, linked to macroeconomic stability (Đorđević & Veselinović, 2010). This macroeconomic stability has disappeared. The savings rates were below the investment rate, the accumulation was mostly imported, while the financing of uncovered consumption and investments was mainly done through borrowing. One of the main limiting factors of development is reliance on foreign savings (Veselinović & Majojević, 2016). Today, it is a general case, primarily in developing countries (including the countries of the Western Balkans) that their investments exceed the domestic accumulation and the autonomous inflow of foreign capital and that this difference is not covered by the compensatory movement of long‐term foreign capital (Radević, Stojadinović‐Jovanović, & Dašić, 2016). The socio‐economic reality of the countries of the Western Balkans was characterized by a high unemployment rate, a high inflation rate, high public debt, a budget deficit, a high level of external debt, a high percentage of the poor population (Đorđević & Lojanica, 2016). Poor privatization processes, war events, the reduction of economic potential, low level of education, widespread corruption, the crisis of the value system, the moral crisis, are all factors that have negatively affected the economy and the lives of the people of the Western Balkans. Bearing in mind the problems that the countries of the Western Balkans faced, we think that the current positions on the HDI ranking list are not at all underestimated.

The experiences of the developed countries point to the conclusion that, in addition to economic stability and growth in production, the requirement of faster economic development is an improvement of the conditions of education and literacy of the adult population (Kulić, Milačić, & Đurić, 2015). Knowledge is a mechanism for raising people from poverty, increasing living standards and promoting economic growth (UN, 2017). Modern society is changing, and education, therefore, needs to be focused on meeting new needs and challenges (Martin, 2016).

Because of the importance of education, many poorer countries have to find ways to adjust their budgets to allocate more money for education. In the absence of their resources, they must turn to international sources (Tostensen, 2007). UNICEF, UNDP, and UNESCO have limited resources to distribute this type of assistance, and therefore are unable to change the modus operandi of their consultants significantly. Insufficiency and lack of education is a severe problem for human development as a whole because it limits the potential for community growth in the income and education dimensions of the HDI.

Thus, poverty reduction, modernization of health infrastructure, improvement in investment in education, increased information literacy rate, stable economic growth are all factors that need to be done to enable better and faster human development, which will result in the growth of HDI (RESI, 2018).

## 5 CONCLUSION

The concept of human development had not changed since 1990 when it was also defined in the first Human Development Report. It has remained focused on the lives, freedoms, and abilities of people. The success in the advancement of human development must be seen through the lives of people living and the skills they have. By analyzing the HDI, we conclude that among the 189 countries observed there are significant differences in the level of Life expectancy at birth, Mean Years of Schooling and Gross national income (GNI) per capita. It does not necessarily mean that countries with the maximum value of certain factors constituting the HDI have a higher HDI value. This is because HDI represents the geometric mean of all three elements that together make up HDI. In the period 1990–2017, at the global level, we have positive HDI growth, as a result of positive movements of all elements. As for the countries of the Western Balkans, they are in the group High Human Development and High Human Development, which is not a minor result given the crisis year at the end of the 20th and the beginning of the 21st century.

All the countries of the Western Balkans have a permanent and mild, but also a continuous increase in HDI indicators, which will lead to further progress in human development. In order to ensure the comprehensive growth of all HDI components, the countries of the Western Balkans must continue to adopt global strategies and laws, realistic action plans, roadmaps for their implementation and the use of knowledge that encompasses a set of skills, competencies, and interests aimed at expanding people's choices and general welfare.

For future investigations of different factors influence on the HDI, there is need for more advanced approach and techniques like fuzzy systems or artificial neural networks which has capabilities of multivariable optimization with different parameters (Mohammadhassani, Saleh, Suhatril, & Safa, 2015; Sadeghipour Chahnasir et al., 2018; ; Sedghi et al., 2018; Toghroli et al., 2018; Toghroli, Mohammadhassani, Suhatril, Shariati, & Ibrahim, 2014).

## CONFLICT OF INTEREST

None declared.

## AUTHOR CONTRIBUTION

Boban Dasic did data analysis, Zeljko Devic did analysis, Nebojsa Denic did literature survey, Dragan Zlatkovic did literature review, Ivana D. Ilic performed analysis, Yan Cao did discussion, Kittisak Jermsittiparsert performed literature review, Hiep Van Le performed literature review

### Peer Review

The peer review history for this article is available at https://publons.com/publon/10.1002/brb3.1755.

## Data Availability

The data that support the findings of this study are available from the corresponding author upon reasonable request.
